# On Methods for the Measurement of the Apelin Receptor Ligand Apelin

**DOI:** 10.1038/s41598-022-11835-z

**Published:** 2022-05-11

**Authors:** Peter Janssens, Henriette de Loor, Jean-Paul Decuypere, Rudi Vennekens, Catherine Llorens-Cortes, Djalila Mekahli, Bert Bammens

**Affiliations:** 1 PKD Research Group, Laboratory of Pediatrics, Department of Development and Regeneration, KU Leuven, Leuven, Belgium; 2 Department of Nephrology and Arterial Hypertension, Universitair Ziekenhuis Brussel (UZ Brussel), Vrije Universiteit Brussel, Brussels, Belgium; 3 Department of Microbiology, Immunology and Transplantation, Nephrology and Renal Transplantation Research Group, University Hospitals Leuven, KU Leuven, Herestraat 49, 3000 Leuven, Belgium; 4 Laboratory of Ion Channel Research, Department of Cellular and Molecular Medicine, Biomedical Sciences Group, KU Leuven, Leuven, Belgium; 5 Laboratory of Central Neuropeptides in the Regulation of Body Fluid Homeostasis and Cardiovascular Functions, Center for Interdisciplinary Research in Biology, Collège de France, INSERM U1050/CNRS UMR 7241, Paris, France; 6 Department of Pediatric Nephrology and Organ Transplantation, University Hospitals Leuven, Leuven, Belgium; 7 Department of Nephrology, Dialysis and Renal Transplantation, University Hospitals Leuven, Leuven, Belgium

**Keywords:** Mass spectrometry, Diagnostic markers, Predictive markers, Prognostic markers

## Abstract

Apelin exists in many isoforms, both in the circulation and in specific tissues. Apelin peptides have a short half-life but preservation before measurement is scarcely studied. Reproducible mass spectrometry methods to specifically measure a broad range of apelinergic peptide isoforms are currently lacking. A sample protocol to conserve apelinergic peptides in the preanalytical phase and a high-performance liquid chromatography–tandem mass spectrometry (HPLC–MS/MS) method to measure apelinergic isoforms was developed. Apelin was measured in plasma. For validation, human embryonic kidney (HEK) cells transfected with cDNA for preproapelin were used. Results were compared with a validated radioimmunoassay (RIA) method. Acidifying plasma to pH 2.5 improves post-sampling stability of apelin. HPLC–MS/MS was unable to detect apelin isoforms in plasma of healthy volunteers (n = 16) and chronic kidney disease patients (n = 4). RIA could detect apelin in concentrations between 71 and 263 fmol/l in 10 healthy volunteers. An optimized preanalytical protocol was developed. A sensitive and specific HPLC–MS/MS method failed to detect apelin in human plasma. Apelin-36 was detected in HEK cells transfected with cDNA for preproapelin. Currently, RIA with relatively selective antibodies is the best alternative for the measurement of apelin but novel sensitive and specific methods are needed.

## Introduction

Apelin is a recently discovered peptide hormone with an important role in water homeostasis and cardiovascular and renal physiology and diseases^1^. It is part of the apelinergic system (AS) that consists of two highly conserved peptide ligands, apelin and apela, and a G-protein-coupled apelin receptor (AR). The AS is widely distributed and plays a pleiotropic but essential role in various physiological and pathological processes. Apela is extensively expressed during gastrulation, Refs.^2–4^ while apelin is only expressed from the end of gastrulation^5^. Apela is downregulated during human embryonic stem cell differentiation. Hence, while apelin and the AR are broadly expressed in adult rats, mice, and humans^6^, apela has a more limited distribution in mature tissues^7^.

**Figure 1:**
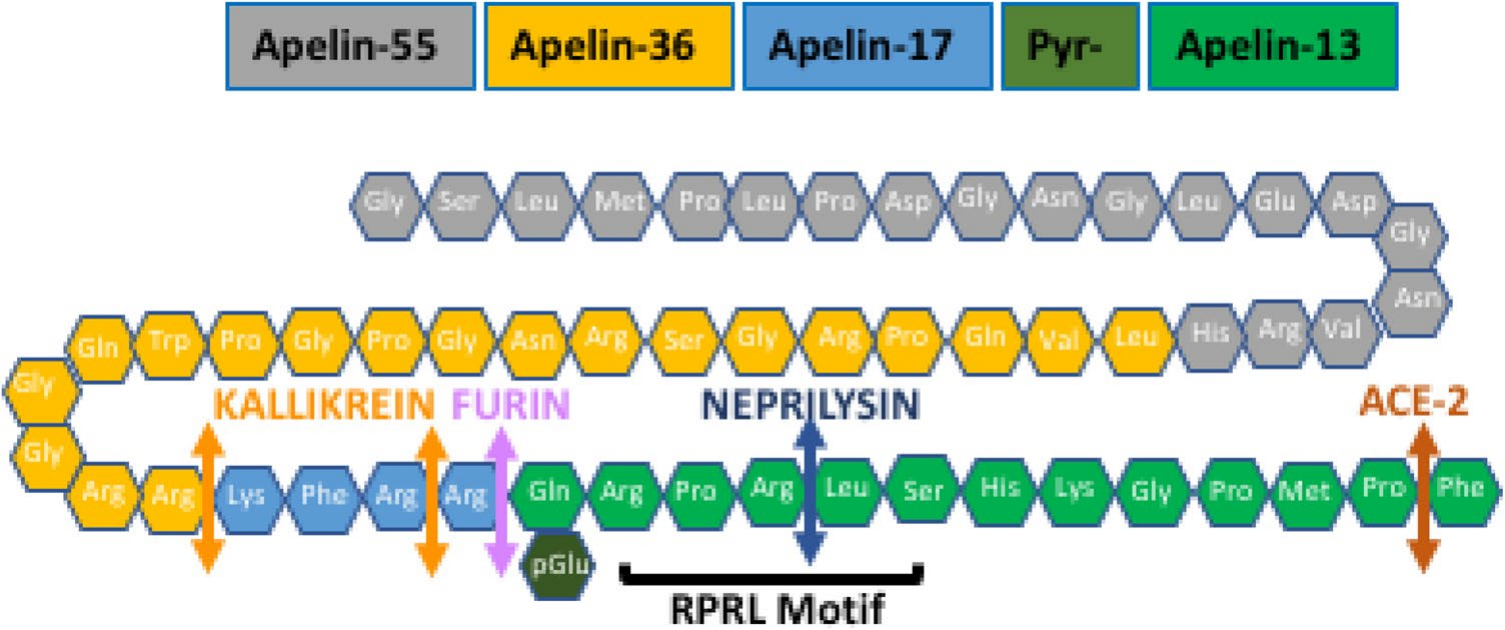
Structure of proapelin. Selective representation of the best characterized peptide fragments. Apelin-55 is processed to several active and inactive isoforms with a short half-life that have not been fully characterized. *ACE-2* angiotensin converting enzyme 2; *neprilysin* neutral endopeptidase 24.11.

Apelin is expressed as a 77–amino acid precursor, and the C-terminal 17 residues are completely conserved in mammalians. Enzymatic processing (overviewed in Table 1) results in several active C-terminal fragments named apelin-n, specifying that a given isoform has “n” amino acids, all sharing the same 12 C-terminal residues that contain the receptor-binding site (Fig. 1). Apelin-55 (named proapelin), -36, -17, -13 and its pyroglutamated form (pyr-13), and -12, are considered the main isoforms and can activate the AR^8–10^. However, recent evidence reveals that various enzymatic processes are likely to be involved in apelin processing and many fragments, both active and inactive, lacking various numbers of N-terminal and C-terminal residues have been detected^11,12^, indicating that the list of “conventional” isoforms above is probably too restrictive. In addition, while pyr-13 apelin and apelin-17 have been determined to be the major isoforms of apelin in the circulation^8,9,13^, their relative contribution remains uncertain and potentially dependent on the study population. Moreover, the relative distribution of isoforms in other biological specimens is still largely unknown. Putative tissue-specific distribution and stability patterns in addition to biased signaling of individual fragments strongly suggest that the measurement of individual apelin fragments will be crucial to gain further insights in the field.

**Table 1:** Enzymatic processing of apelin.

Enzyme	Conversion	Effect	Refs.
*Furin (PCSK3)*	Apelin 55 and 36 directly to apelin 13; Apela 32 to apela 11	Active apelin 13 fragments	^25,26^
*Angiotensin converting enzyme-2 (ACE-2)*	Hydrolysis of C-terminal phenylalanine of all apelin isoforms. Pyr-apelin-13 is more susceptible to ACE-2 degradation than apelin-17	Both fully active and reduced activity fragments	^27^
*Neprilysin*	Cleavage of “RPRL” motif	Inactivation	^28^
*Kallikrein*	Cleaves the 3N-terminal amino acids of apelin-17	Apelin-14 with decreased hypotensive effect	^29^
*Prolyl carboxypeptidase*	Cleaves apelin 36, 17 and pyr-apelin 13	Similar to ACE-2 effect	^30^

Other, yet unidentified enzymatic processes are most likely involved.

Plasma apelin levels are reported mainly using commercial, typically competitive, enzyme immunoassays. These assays use polyclonal antibodies raised against various synthetic peptide fragments, raising questions about their specificity. Moreover, as the conserved C-terminal domain is often targeted, it remains unknown which apelin fragments are measured, and most probably a plethora of fragments is detected. This can partially explain the wide variability of at least a factor 100 in reported plasma concentrations obtained with these assays. Alternative methods combining high-performance liquid chromatography (HPLC) with radioimmunoassay (RIA) detection have been described to improve the methodology of apelin measurements and identify the different isoforms. In one such method, based on rabbit polyclonal antibodies directed against apelin 17, a relative specificity of the antibodies for apelin 13,17 and 36 fragments was confirmed using iodinated apelin-13 and 17 as tracers. Reactivity decreased with loss of amino acids from the N-terminal part of apelin-17 and was negligible with C-terminally truncated fragments of apelin 17 and various other bioactive peptides, including angiotensin II, angiotensin III, neuropeptide Y, and AVP^9,14,15^. Using this method, apelin 17, pyroglutamated apelin 13 and apelin 36 (at a much lower concentration) could be detected in plasma of healthy volunteers in concentrations of about 250–500 fmol/ml^9,16^. However, only a limited number of isoforms have been evaluated with RIA. As these methods make use of polyclonal antibodies, inherent issues of specificity cannot be excluded. Lastly, detection of naturally circulating apelin peptides with mass spectrometry (MS) has been attempted. With this approach, one group was able to detect pyroglutamated apelin at a level of a few pg/mL in samples of healthy subjects^8,11^, while others were unable to detect any native apelin^12,17^.

In addition, the very short half-life of the apelinergic peptides, probably a few minutes^12,18^, further complicates the measurement. While several methods, including acidification, cooling and protease inhibitors have been used, the most reliable way to preserve apelinergic peptides before measurement has yet to be determined.

**Figure 2:**
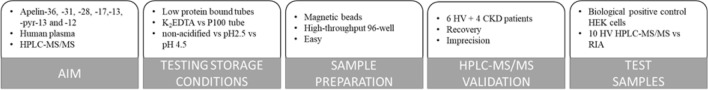
Schematic overview of the experimental design. *HV* healthy volunteer, *CKD* chronic kidney disease.

There is increased interest in the role of apelin in physiology and pathophysiology and stable apelinergic analogs have been generated. Research in the field is hampered by the lack of easily accessible, reliable and reproducible measurement methods. In the present study (overviewed in Fig. 2), we aimed to optimize the conservation of the apelinergic peptides in the preanalytical phase and developed an improved high-performance liquid chromatography–tandem mass spectrometry (HPLC–MS/MS) method to measure specific apelin isoforms in human plasma samples. For validation, a biological positive control from human embryonic kidney (HEK) cells transfected with apelin was used. Finally, plasma apelin of healthy volunteers measured by a validated RIA method was compared with the results obtained with the HPLC–MS/MS method.

## Materials and methods

### Materials

Apelin-36, -17, -13, pyr-13, -12 were purchased from CPC Scientific (San Jose, CA, USA). Apelin-31, -28 and for additional conformation -36, 13, pyr-13 and -12 were purchased from Phoenix Pharmaceuticals (Burlingame, CA, USA). For internal standards, stable isotope-labeled (SIL) peptides, synthesized by CPC Scientific (San Jose, CA, USA), were used. Stable isotope-labeled proline residues ([^13^C_5_; ^15^N]Pro) were incorporated into these peptides on proline positions: position 2 and 9 (apelin-12), 3 and 10 (apelin-13 and pyr-13), 7 and 14 (apelin-17), and 13, 26 and 33 (apelin-36). This results in a mass shift of 6 Da for every proline exchanged. For apelin-31 and -28, SIL apelin-36 was used for the internal standard.

Acetonitrile and formic acid were MS grade and purchased from Biosolve BV (Valkenswaard, the Netherlands) and isopropyl alcohol with analytical grade from Fisher Scientific (Geel, Belgium). Purified water was prepared using a Milli-Q (MQ) water system from Millipore (Billerica, MA, USA). Radioimmunoprecipitation assay (RIPA) lysis buffer containing 10 mM sodium phosphate (pH 7.5), 150 mM NaCl, 1.5 mM MgCl_2_, 0.5 mM DTT, 1% Triton X-100, and protease and phosphatase inhibitor cocktails from Roche (Basel, Switzerland).

### Preparation of analytical standards

An internal standard mix solution in MilliQ water/acetonitrile (90/10) + 0.1% formic acid was prepared with a final concentration of 20 µg/ml for apelin-36 and 2 µg/ml for apelin-17, -13, pyr-13 and 12. The internal standard mix was aliquoted in low protein bound binding tubes and stored at − 80 °C.

Apelin isoform stock solutions were prepared in MilliQ water/acetonitrile (90/10) + 0.1% formic acid, aliquoted in low protein bound binding tubes and stored at − 80 °C. Daily, freshly prepared 10-point calibration covering the desired range were prepared, and finally spiked before sample preparation into a blanc (free of endogenous apelin)plasma pool of healthy volunteers. The endogenous apelin was assumed to be degraded by incubating this healthy plasma pool overnight at 37 °C.

For apelin-36, -31 and -28, only 6 of the 10 points of the calibration were used over a final concentration range of 1400–40 pg/ml. For apelin-17, -13, pyr-13 and -12, 10-point calibration curves were used with a final concentration range of 500–1 pg/ml.

### Preanalytical considerations—storage conditions

**Figure 3:**
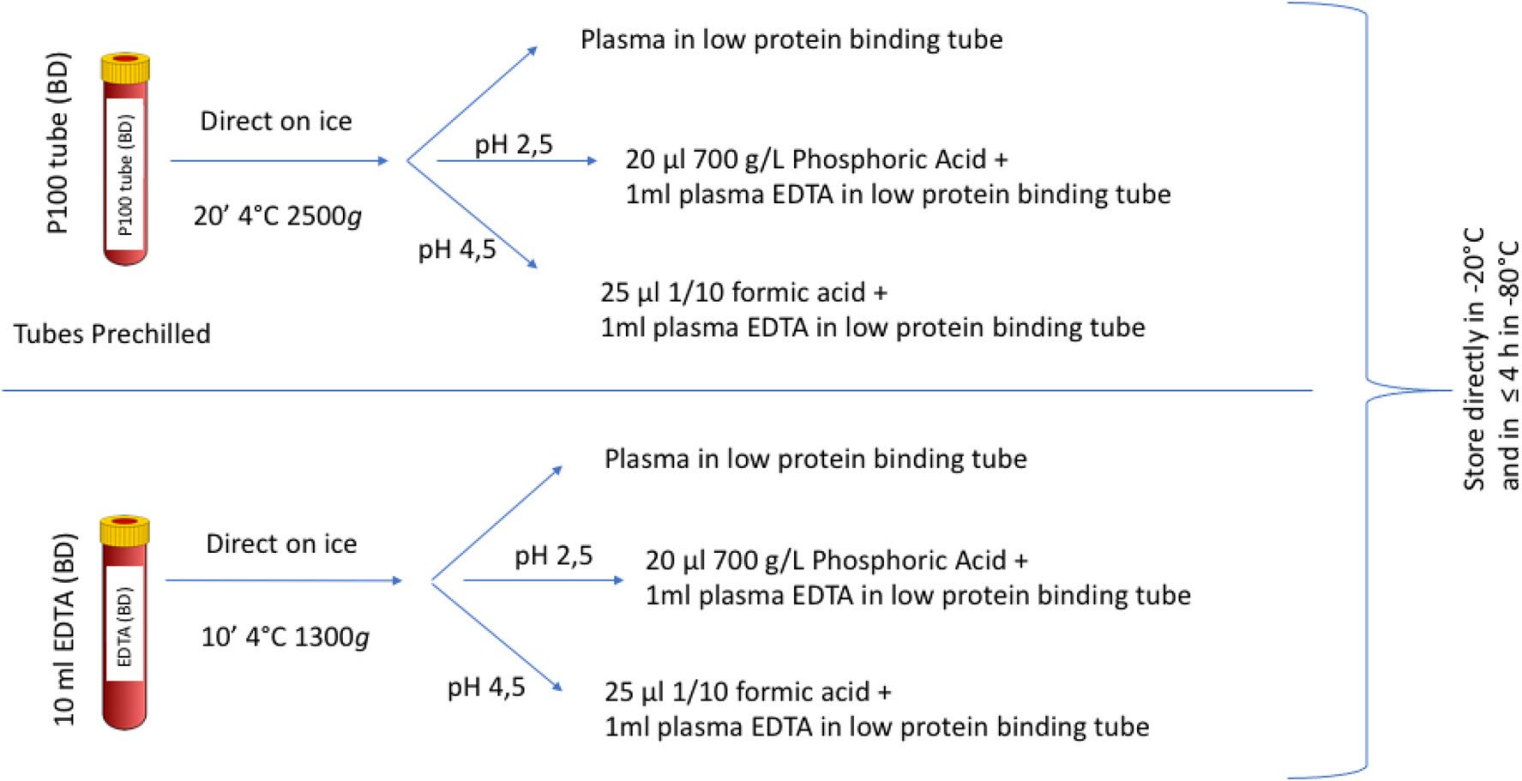
Overview of preanalytical conditions. Samples were immediately cooled on ice and processed within 1 h. Both standard K_2_EDTA tubes and P100 prechilled tubes containing a protease inhibitor cocktail and mechanical separator were used. The standard K_2_EDTA was centrifuged for 10 min 1300 × *g* at 4 °C, while the P100 tube was centrifuged for 20 min 2500 × *g* at 4 °C, according to the manufacturers’ instructions. After separation, plasma was untreated or acidified to either pH 2.5 or 4.5 by adding the plasma to low protein-binding tubes pre-filled with either phosphoric acid or formic acid. The plasma samples were stored directly at − 20 °C and transferred within 4 h at − 80 °C.

In view of the putative very short half-life of the apelin peptides, variations in measured concentrations driven by variations in protein degradation should be expected. We aimed to minimalize degradation after sampling with a preanalytical protocol summarized in Fig. 3, including immediate cooling and rapid processing of samples. In addition to a previously described plasma acidification step to pH 4.5, which provide stability for the apelin fragments -36, -17, -13^8^, we added acidification to pH 2.5 in an attempt to improve the stability of all fragments. However, acidification can be applied only after separation by centrifugation of whole blood into a plasma and a cell compartment, because acidification of whole blood would lead to hemolysis and coagulation, rendering the blood samples unsuitable for further measurements. Therefore, we used protease inhibitor cocktail-containing K_2_EDTA-coated P100 blood tubes and a mechanical plasma separator (366422 BD Biosciences, San Jose, CA, USA)^19^. The aim of the protease inhibitor cocktail is to inactivate proteolytic degradation by enzymes present in whole blood; the mechanical separator prevent contact with cell-bound enzymes, both theoretically limiting enzymatic degradation of apelin during the several minutes between blood collection in the tube and completed plasma separation and acidification.

Lastly, as adsorption of proteins and peptides to polymer surfaces can contribute to sample loss during storage and handling^20^, low protein binding tubes (BIOplastics B71072U) and low retention pipet tips (Eppendorf, epT.I.P.S LoRetention) were used during all sample manipulations.

### Apelin transfected human embryonic kidney cells

HEK 293T cells were grown to 80% confluence. 400 µl Opti-MEM Reduced-Serum Medium (Gibco, Thermo Fisher Scientific, Waltham, MA, USA) and 24 µl TransIT-293 Reagent (Mirus, Madison, WI, USA) were vortexed and incubated for 20 min at room temperature. 8 µg plasmid DNA (Myc-DDK tagged human apelin clone, RC205832, OriGene, Rockville, MA) was added and incubated for 15 min. Cell culture medium was removed and 8 ml fresh medium and the transfection mixture was added. Cells were incubated at 37 °C and grown to 70 to 90% confluence.

**Figure 4:**
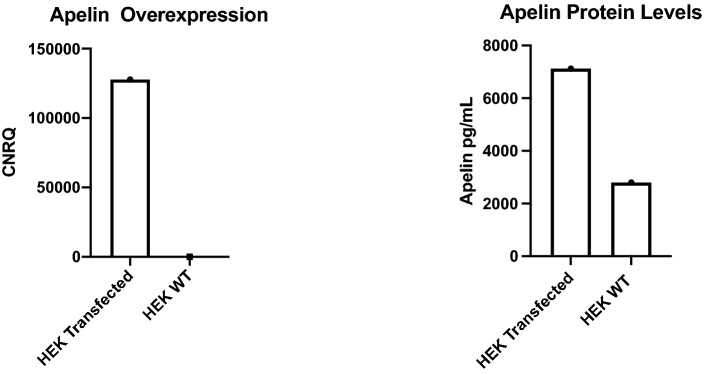
Left: Transfection of HEK293 cells with human preproapelin cDNA confirmed by qPCR. Right: apelin-36 levels in HEK WT and HEK transfected as measured with MS.

Successful transfection was first confirmed with quantitative polymerase chain reaction (qPCR) (Fig. 4). For qPCR, ribonucleic acid (RNA) was extracted from the cell pellets using the RNeasy Mini Kit (Qiagen, Hilden, Germany) according to the manufacturer’s protocol. The concentration and purity of the extracted RNA is determined using the Nanodrop Spectrophotometer (ND-1000, Isogen Life Science). Complementary deoxyribonucleic acid (cDNA) is then synthesized using the High-Capacity cDNA Reverse Transcription Kit (Applied Biosystems, Waltham, MA, USA), according to manufacturer’s protocol. qPCR was performed using the Applied Biosystems StepOnePlus Real-Time PCR systems (Thermo Fisher Scientific, Waltham, MA, USA). with apelin primer probe set Hs00175572_m1 (Thermo Fisher Scientific, Waltham, MA, USA), and results were referenced to beta-actin (ACTB) and glyceraldehyde 3-phosphate dehydrogenase (GAPDH) expression. Cells were harvested and homogenized in a RIPA lysis buffer, acidified to pH 2.5 with formic acid. Protein concentration was determined by bicinchoninic acid (BCA) assay (Thermo Fisher Scientific, Waltham, MA, USA). Cell medium was collected, acidified to pH 2.5 with formic acid, immediately snap frozen and stored at − 80 °C.

### Sample preparation

For experiments, plasma samples were defrosted to 4 °C. Dynabeads MyOne carboxylic acid magnetic beads (Thermo Fisher Scientific, Waltham, MA, USA) were used for sample cleanup, with the aim of retaining apelin peptides on the beads while allowing for removal of impurities from the plasma samples. These beads were washed twice with MilliQ water (using fivefold the magnetic beads volume of water) before use. For each plasma sample, 40 µl magnetic beads is needed. 40 µl magnetic beads were resuspended in 25 µl 100 mM sodium phosphate buffer pH 7 for preparation of plasma samples without acidification and at pH 4.5 and 200 mM sodium phosphate buffer pH 7 for preparation of plasma samples stored at pH 2.5.

Once the beads solution was ready, sample cleanup was performed in 700 µl QuanRecovery 96-well plates (Waters, Antwerp, Belgium). 250 µl of centrifuged (3 min 16,000×*g* at 24 °C) sample (plasma whether or not acidified or RIPA buffer pH 2.5 lysates from HEK cells) was loaded together with 10 µl internal standard solution and 20 µl calibration point or background calibration solution (MilliQ water/acetonitrile (90/10) + 0.1% formic acid). This was done for similarity to the calibration standards. Next, 250 µl of 100 mM (plasma stored without acidification and at pH 4.5) or 200 mM (plasma stored at pH 2.5) sodium phosphate buffer pH 7 and 25 µl bead solution (in the same molarity of buffer) was added. Plates were put on an orbital shaker for 5 min at room temperature at 800 RPM. Subsequently, the plates were placed on a 96-well magnetic stand (Thermo Fisher Scientific, Waltham, MA, USA) for 3 min and the supernatant was removed. After removing the magnet, the magnetic beads were washed twice, by resuspending in 250 µl MilliQ water, orbital shaking for 2 min at 800 RPM at room temperature and supernatant was removed by placing on the magnetic stand for 2 min. Desorption of peptides was achieved by adding twice 25 µl MilliQ water/acetonitrile (60/40) + 1% trifluoroacetic acid (TFA), orbital shake for 2 min at room temperature at 500 RPM and placing on the magnetic stand for 1 min, and each time transfer of the 25 µl fractions and mixing them together to a new 700 µl QuanRecovery 96-well plate. Finally, plates were covered with a capmat and put on the orbital shaker for 2 min at room temperature at 800 RPM. Next, 96-well plates were centrifuged for 3 min at 24 °C and 2000×*g*, before transfer to the chromatographic system.

### HPLC–MS/MS method

HPLC–MS/MS was performed using an Acquity H Class UPLC with a Xevo TQS tandem mass spectrometer (Waters, Antwerp, Belgium). An injection volume of 15 µl was used and chromatographic separation was performed on a Polaris 3 C18-A column (100 × 2.0 mm; 3.0 µm particle size) with a Polaris 3 C18-A MetaGuard pre-column (10 × 2.0 mm; 3.0 µm particle size) (Agilent, Santa Clara, CA, USA). The mobile phase, delivered at a flow rate of 0.35 ml/min at 50 °C, was a gradient of 0.1% formic acid in MilliQ water (A) and 0.1% formic acid and 0.1% isopropyl alcohol in acetonitrile (B). The total run time was 15 min and the gradient was as follows: start with a constant of 5% B for 2 min, directly increase to 13,5% B and maintain for 3 min, gradual increase to 70% B in 1 min, maintain 2 min, directly reintroduce the initial 5% B and equilibrate the system 7 min before the next injection.

Ionization was achieved using electrospray positive ionization mode (ESI+). Nitrogen was used as nebulization and desolvation gas and argon as collision gas. Except for SIL internal standards, two multiple-reaction monitoring (MRM) transitions were used for each peptide. The MRM transitions, cone voltage and collision energy were empirically determined for each individual compound (Table 2). Other parameter settings were as follows: source temperature of 150 °C, desolvation temperature of 650 °C, desolvation gas flow of 1050 L/hr, cone gas flow of 150 L/hr and capillary voltage of 1.0 kV. Data acquisition and processing was performed using MassLynx software, version 4.2 (https://www.waters.com/waters/en_US/MassLynx-MS-Software/nav.htm?locale=en_US&cid=513662).

**Table 2:** MRM transition and mass spectrometer settings.

	Q1 (m/z)	Q2 (m/z)	Cone voltage (V)	Collision energy (V)
Apelin-36	525.5 (8+)	583.9	55	14
		637.4	55	17
([^13^C_5_,^15^N]Pro^13,26^^,33^) apelin-36	527.3 (8+)	586.5	55	14
Apelin-31	515.7 (7+)	596.0	45	14
		607.5	45	14
Apelin-28	551.3 (6+)	626.8	45	14
		522.5	45	16
Apelin-17	428.6.6 (5+)	469.7	40	12
		457.8	40	14
([^13^C_5_,^15^N]Pro^7,14^) apelin-17	431.0 (5+)	472.7	40	12
Apelin-13	388.7 (4+)	430.2	40	12
		414.2	40	14
([^13^C_5_,^15^N]Pro^3,10^) apelin-13	391.6 (4+)	434.2	40	12
Apelin-pyr-13	384.4 (4+)	424.6	40	12
		408.6	40	14
([^13^C_5_,^15^N]Pro^3,10^) apelin-pyr-13	387.3 (4+)	428.5	40	12
Apelin-12	356.7 (4+)	387.6	40	10
		371.6	40	12
([^13^C_5_,^15^N]Pro2^2,9^) apelin-12	359.6 (4+)	391.5	40	10

### Study population

For the method development, 6 healthy volunteers with mean age (SD) 39.60 (14.96), male/female ratio 3/3 and 4 subjects with chronic kidney disease (CKD) with mean age (SD) 49.22 (4.59), male female ratio 3/1 were included. For final validation, 10 healthy volunteers with mean age (SD) 25.17 (7.69) and male/female ratio 6/4 were tested. The characteristics of the CKD subjects are summarized in Table 3. The reason for studying subjects with CKD was that we assumed for theoretical reasons that the aquaretic peptide apelin could be increased in this population, compared to healthy subjects. The samples were treated as discussed in the preanalytical considerations—storage conditions-sections. Informed consent was obtained from all participants. The study was performed according to the Declaration of Helsinki and approved by the ethics committee of the University Hospitals Leuven (S60070 and S62008).

**Table 3:** Characteristics of CKD patients.

Subject	Age (years)	Sex	eGFR (ml/min/1.73 m^2^)	Underlying condition
1	44.6	Male	24	ADPKD
2	54.1	Male	26	ADPKD
3	45.8	Male	38	Membranous nephropathy
4	47.8	Female	39	Nephrocalcinosis

*ADPKD* autosomal dominant polycystic kidney disease, *eGFR* estimated glomerular filtration rate according to CKD-EPI.

## Results

Based on the literature the following peptides were selected as a target for measurements: apelin-36, -17, -13, pyr-13 and -12. After examining the Universal Protein Resource (UniProt) database, we additionally selected apelin-31 and -28.

### Method validation—analysis of endogenous peptides in plasma samples

The internal standard mix solution and apelin isoform stock solutions were prepared in MilliQ water/acetonitrile (90/10) + 0.1% formic acid. This solution was taken on the basis of the two stability tests. Apelin-36, -17, -13, pyr-13 and -12 were individually resolved in different conditions (MilliQ water, MilliQ water + 1% formic acid, MilliQ water + 0.1% formic acid, MilliQ water/acetonitrile (95/5) + 0,1% formic acid and MilliQ water/acetonitrile (90/10) + 1% formic acid). The area obtained by direct injection of the solutions into the HPLC–MS/MS system (no sample preparation) is used for the comparison of the stability test. The first stability test is the evaluation of 6 days in the refrigerator at 4 °C (injection at baseline “fresh”, day 1, 2 and 6). The second stability test of the solutions was examined before and after freeze/thaw cycle (solution were stored at − 80 °C) with an injection at baseline “fresh” and after 1, 2 and 3 freeze/thaw cycles. The results are reported in Supplementary Figs. S1 and S2.

Because we were unable to detect endogenous apelin peptides in the plasma of 6 healthy volunteers and 4 CKD patients during the initial experiments, we determined apelin peptide recovery by adding a fixed concentration of the synthetic peptides both at the beginning (before adding magnetic beads) and in an identical sample at the end (directly into the eluent after adsorption from the magnetic beads) of sample preparation. The difference in the analyte recovered determines the recovery of the sample preparation.

We determined spiking recovery on 6 different K_2_EDTA plasma samples from healthy volunteers, each stored by the 3 different conditions: plasma without acidification, plasma at pH4.5 and plasma at pH2.5.

Recovery of apelin-36, -31, -28, -17, -13, -12 was within the acceptable range for all preanalytical conditions. Recovery of apelin-pyr-13 was between 60 and 76% for plasma stored at pH 2.5 and between 50 and 63% for plasma stored without acidification and between 50 and 71% for plasma stored at pH 4.5 (Table 4). These satisfying recovery rates allowed us to validate the sample preparation protocol and rules out that the failure to detect endogenous peptide in plasma was related to a loss of apelin during sample preparation.

**Table 4:** Recovery experiment based calculated on area (n = 6).

Peptide	Recovery % (area) Mean ± stdev		
	Plasma	Plasma pH 4.5	Plasma pH2.5
Apelin-36	83.34 ± 3.66	83.15 ± 11.87	75.02 ± 7.46
Apelin-31	73.15 ± 11.09	86.96 ± 9.58	89.29 ± 8.60
Apelin-28	81.87 ± 13.10	84.10 ± 10.84	95.50 ± 10.19
Apelin-17	80.51 ± 5.25	80.64 ± 7.53	75.30 ± 3.65
Apelin-13	75.80 ± 7.14	76.66 ± 7.60	75.33 ± 5.44
Apelin-pyr-13	55.28 ± 4.80	60.63 ± 7.95	70.17 ± 6.77
Apelin-12	72.50 ± 6.78	79.91 ± 8.59	73.60 ± 4.23

The recovery was determined by adding a fixed concentration of the peptides both at the beginning of sample preparation and at the end.

For precision determination, triple measurements after spiking of synthetic peptides were performed on K_2_EDTA and P100 plasma of healthy volunteers (n = 6), stored in non-acidified condition or stored acidified to pH4.5 and 2.5. The coefficients of variation (CV%) for all conditions were below 9% (Table 5). The chromatograms of each isoform of apelin and their respective internal standards are shown in Supplementary Fig. S3.

**Table 5:** Precision determination.

	Spiked (pg/ml)	K_2_EDTA		P100	
		pH2.5	pH4.5	pH2.5	pH4.5
Apelin-36	1114	4.44	6.75	6.29	7.89
Apelin-31	1114	5.52	4.39	6.25	8.24
Apelin-28	1114	3.82	4.01	5.06	5.41
Apelin-17	384	6.70	7.20	4.96	3.03
Apelin-13	384	2.40	2.60	3.67	4.24
Apelin-pyr-13	384	2.65	2.11	2.66	2.98
Apelin-12	384	5.18	2.30	8.03	2.82

Triple measurements after spiking of synthetic peptides on K_2_EDTA and P100 healthy plasma (n = 6) in non-acidified and acidified to pH4.5 and 2.5. Data is provided as the coefficients of variation (CV%).

### Calibration curves, limit of quantification (LOQ), limit of detection (LOD)

Calibration curves were constructed using peak area ratios of analyte-to-internal standard using 1/X weighted linear regression. Linearity of calibration curves was assumed when the measured concentration of each calibration standard was within 10% of the nominal value, except for the lower limit of quantification (LLOQ) for which a deviation of 15% was considered acceptable. The calibration curves were injected at the start and at the end of each sample set. The limit of detection (LOD) was determined as previously described^21^. Data on the calibration curves, LLOQ and LOD are shown in Table 6.

**Table 6:** Calibration curves information.

Peptide	Calibration range (pg/ml)	Calibration range (fmol/ml)	LLOQ (pg/ml)	LOD (pg/ml)
Apelin-36	1400.0–40.0	333.6–10.4	80.0	40.0
Apelin-31	1400.0–40.0	388.6–12.2	80.0	40.0
Apelin-28	1400.0–40.0	424.0–13.2	80.0	40.0
Apelin-17	500.0–1.0	233.8–0.5	2.0	1.0
Apelin-13	500.0–1.0	322.4–0.6	2.0	1.0
Apelin-pyr-13	500.0–1.0	325.9–0.6	8.0	4.0
Apelin-12	500.0–1.0	352.1–0.7	2.0	1.0

### Stability of synthetic peptides according to the preanalytical condition

Spiking of individual peptides was performed on plasma collected in K_2_EDTA tubes, stored either non-acidified, or acidified to pH 4.5 or pH 2.5 and on plasma collected in P100 tubes, stored either non-acidified, or acidified to pH 4.5 and pH 2.5. The samples were measured both immediately and after 60 min at room temperature, each sample was measured 3 times.

**Figure 5:**
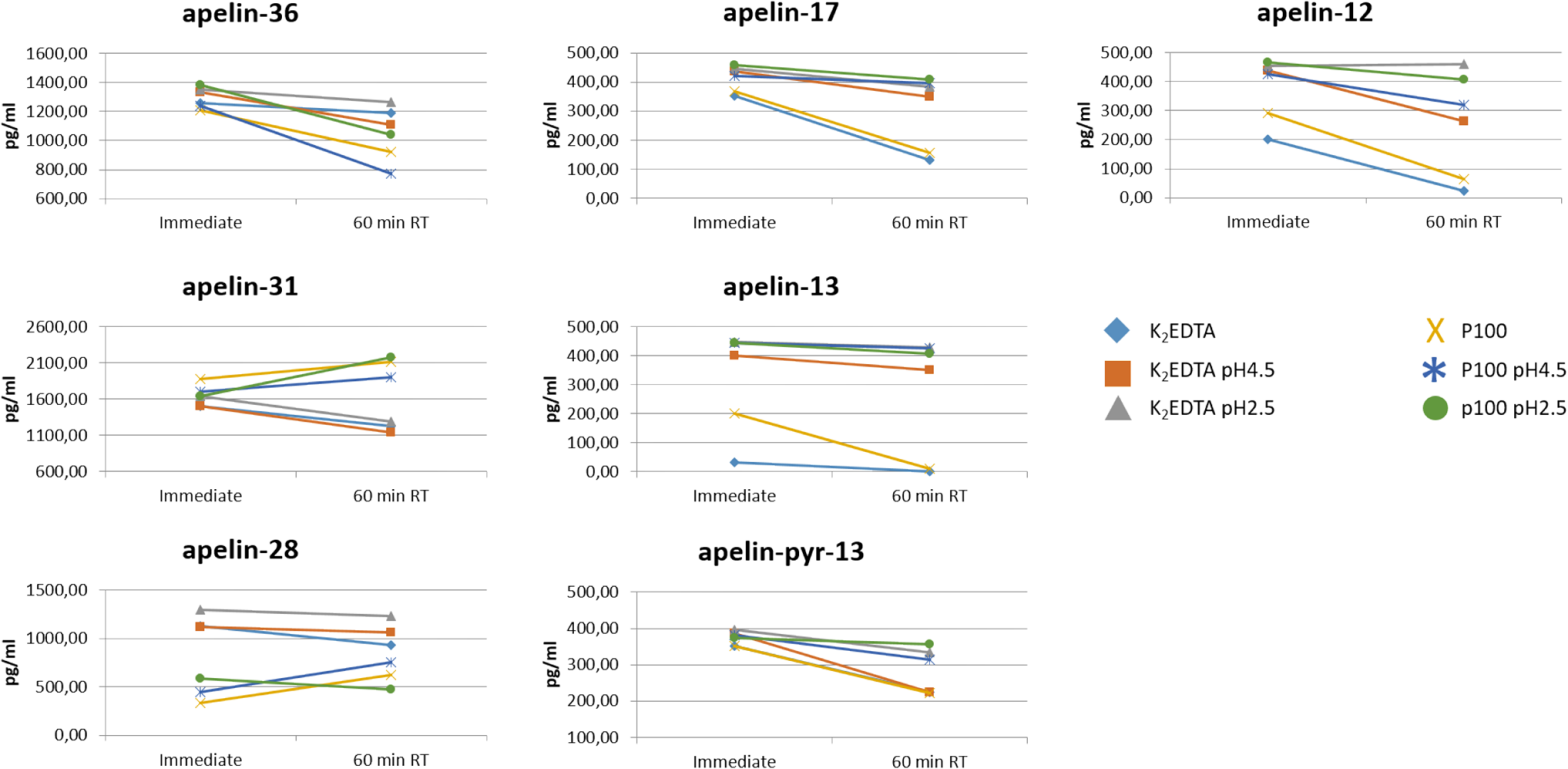
Stability of synthetic peptides under different storage conditions. Apelin-36 exhibits limited degradation after one hour at room temperature in plasma collected in K_2_EDTA tubes and stored non-acidified or stored acidified to pH 4.5 and pH 2.5. Limited degradation is also observed in plasma collected in a P100 tube and stored acidified to pH 2.5. In P100 plasma stored non-acidified and at pH4.5, a higher degradation is observed. P values after Kruskal–Wallis test for the difference between last and first time point: apelin-36: P = 0.0117; apelin-31: P = 0.0841; apelin-28: P = 0.0168; apelin-17: P = 0.0207; apelin-13: P = 0.0318; apelin-pyr-13: P = 0.0079; apelin-12: P = 0.0099. *RT* room temperature.

The results are shown in Fig. 5. Apelin-36 exhibits limited degradation after one hour at room temperature in K_2_EDTA plasma non-acidified, pH 4.5 and pH 2.5 and P100 pH 2.5. In P100 plasma non-acidified and pH4.5 a higher degradation is observed.

Because we observed sometimes an increase in apelin-31 and -28, after incubation in plasma, we suspect that P100 can cause ion suppression during the ionization in the mass spectrometer, inducing measurement errors. Therefore, we cannot draw definitive conclusions on the peptide stabilizing properties of the P100 tube.

We do not observe an influence of pH for apelin-31 in K_2_EDTA plasma, while apelin-28 has a slightly better stability in K_2_EDTA plasma at pH 4.5 and pH 2.5 than in non-acidified plasma.

For apelin-17 and -13, we observe rapid degradation in non-acidified K_2_EDTA and P100 plasma but only minor degradation at pH 4.5 and 2.5.

For apelin-pyr-13, we conclude that pH 2.5 results in the best stability. Similarly, apelin-12 is most stable at pH 2.5. Note that apelin-17, -13 and -12, exhibit immediate degradation in non-acidified conditions, suggesting that acidification should be done as soon as possible. Theoretically, this can be done on whole blood during collection. However, as far as we know there are no blood collection tubes with an acid additive available. Moreover, acidification causes hemolysis, which further complications sample preparation and analysis.

**Figure 6:**
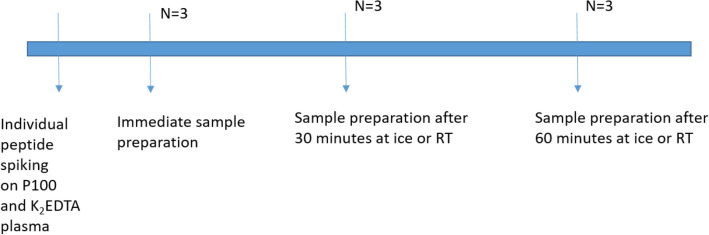
Overview of testing influence of P100 tubes on peptide stability. To test whether the P100 tube protease inhibitor cocktail results in additional peptide stability before blood tubes centrifugation, we spiked apelin-36, -17, -13, -pyr-13 and -12 on K_2_EDTA and P100 plasma. The samples were measured immediately, or after 30 and 60 min on ice or at room temperature.

**Figure 7:**
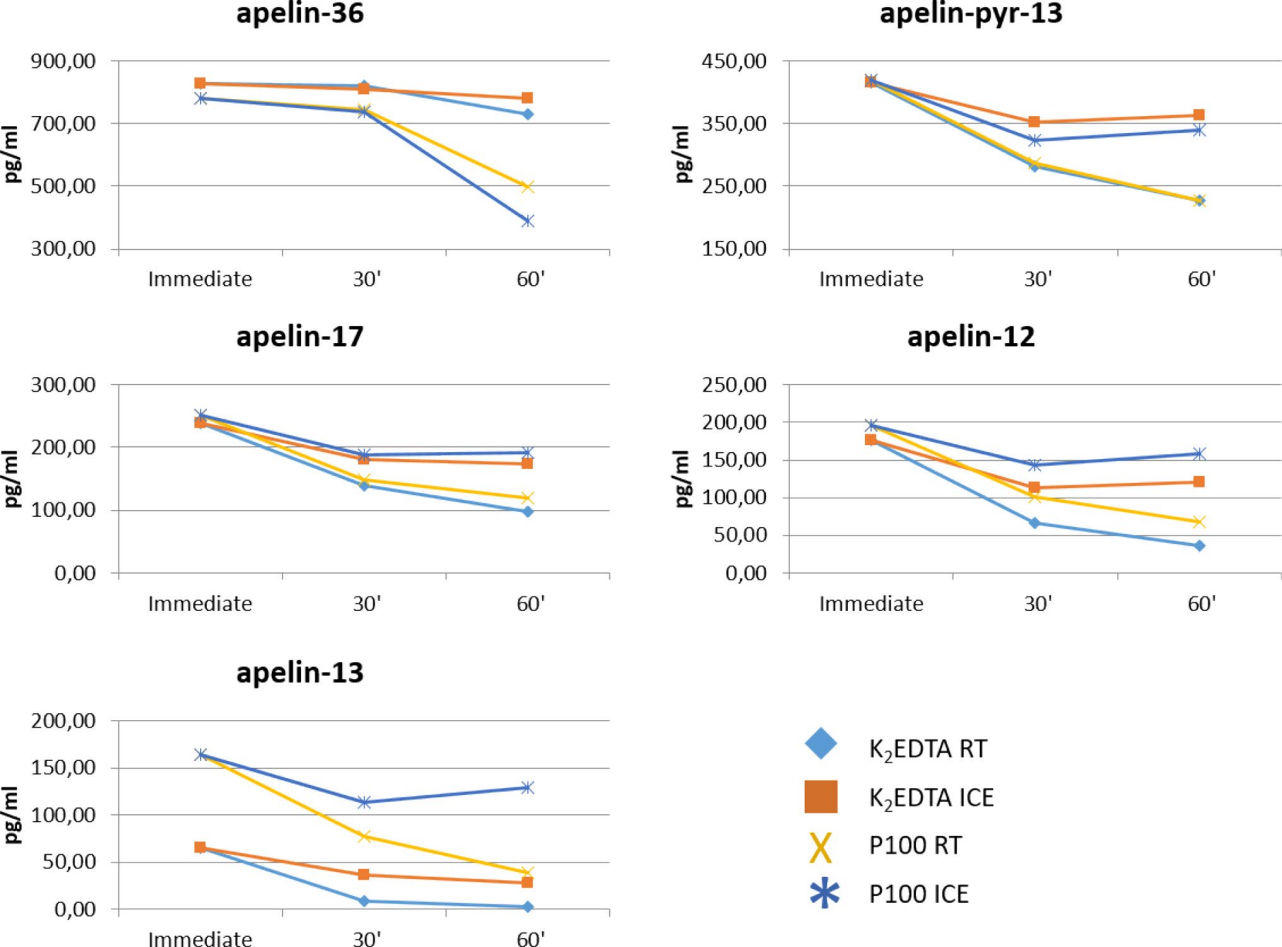
Influence of cooling on ice and P100 protease inhibitor cocktail on peptide stability in whole blood before centrifugation. Cooling with ice has the largest impact on stability, with limited added value of the P100 tube. Only for apelin-13 there a small added value in terms of stability due to the P100 tube was observed. For apelin-36, the P100 tube seems to have a negative influence. P values after Kruskal–Wallis tests for the difference between last and first time point: apelin-36: P = 0.023; apelin-17: P = 0.039; apelin-13: P = 0.270; apelin-pyr-13: P = 0.238; apelin-12: P = 0.135.

To test whether the P100 tube protease inhibitor cocktail results in additional peptide stability before blood tubes centrifugation, we spiked apelin-36, -17, -13, -pyr-13 and -12 on K_2_EDTA and P100 plasma. The samples were measured immediately, and after 30 and 60 min on ice or at room temperature (Fig. 6). All samples were measured in triple. The results are shown in Fig. 7. We conclude that cooling with ice increases stability, with limited added value of the P100 tube.

The general conclusion is that none of the storage methods is able to fully inhibit peptide degradation. Apelin peptide degradation can be significantly inhibited by immediate cooling and centrifugation of blood samples, and by rapidly acidifying plasma to pH2.5.

### Biological positive control: apelin transfected HEK cells

**Figure 8:**
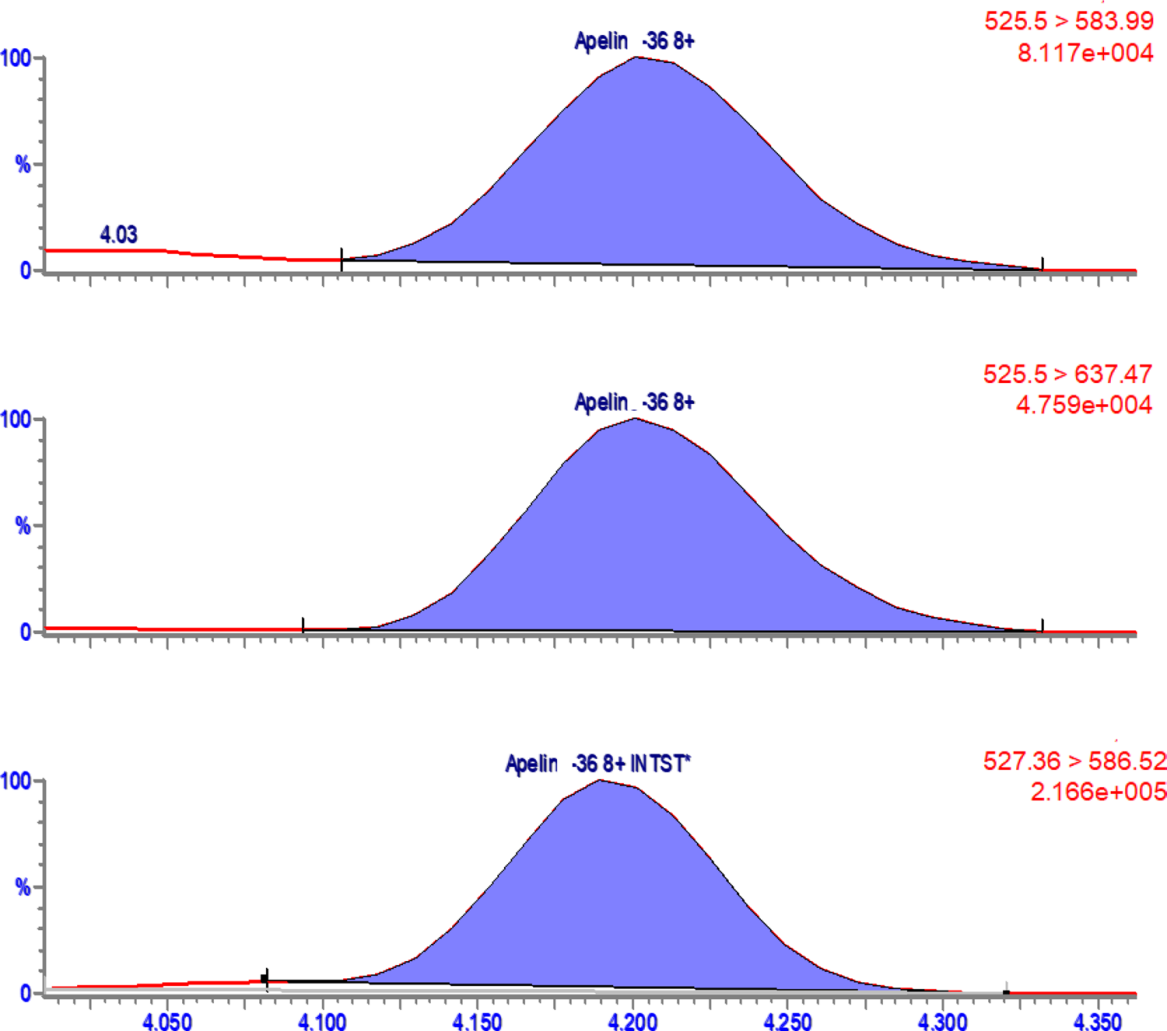
Chromatogram of the two MRM transitions of apelin-36 (the 2 top chromatograms) and its SIL internal standard (bottom chromatogram) in the transfected HEK cells. Additional chromatograms are in Supplementary Fig. S3.

Because we could not detect apelin in human plasma samples during initial experiments, we measured HEK cell samples before and after transfection with apelin, as a biological positive control. On cell culture medium we again did not detect any apelin peptides. In lysates of sham transfected cells, we only observed apelin-36, at concentration of 2797 pg/ml, while the concentration in transfected lysates was 7121 pg/ml (see apelin-36 chromatogram in Fig. 8). It should be noted that this concentration was higher than our highest calibration point, so extrapolation was used.

### Comparison of plasma apelin measured by RIA and MS/MS

Plasma samples of 10 subjects (6 healthy persons and 4 CKD patients) were used for experiments during method development. In addition, plasma samples of 10 healthy volunteers were measured both with the HPLC–MS/MS method as described above and with a previously published and validated RIA method. This RIA method has a detection limit of 6 fmol/ml, and a quantification limit of 12 fmol/ml^9,14,15^. Simultaneously obtained plasma, collected with K_2_EDTA tubes and stored under pH 4.5 and pH 2.5 was used for these measurements. As apparent from Table 7, while the RIA method was able to measure apelin peptides, samples analyzed with HPLC–MS/MS remained below the LLOQ.

**Table 7:** Results biological samples.

	Apelin-IR (fmol/ml)
HV 01	172.54
HV 02	224.56
HV 03	235.12
HV 04	263.01
HV 05	168.05
HV 06	240.51
HV 07	210.03
HV 08	180.05
HV 09	150.60
HV 10	70.98

No individual apelin fragments were detected by HPLC–MS/MS, while the RIA method does detect apelin immunoreactivity (apelin-17 + pyroglutamyl apelin-13 + apelin-36).

*apelin-IR* apelin immunoreactivity, *HV* healthy volunteer.

## Discussion

In this study, we were able to show that rapid cold processing of blood samples and acidification of plasma to pH 2.5 improves the post-sampling stabilization of apelin isoforms and as such has the potential to optimize the detection and quantification of apelin in biological samples. In addition, starting from published methods^8,11^, we optimized a tandem mass-spectrometer to measure specific apelinergic isoforms in the circulation. This study built on scare previously published work on the MS detection of apelin, including that of Mesmin et al.^11^ and Zhen et al.^8^ Compared to the work of Zhen et al., we further optimized the published methods in several aspects: (1) the use low protein binding tubes and material throughout the experiment (2) improved stabilization of endogenous apelin peptides through cooling and acidifying human plasma, (3) added a broad spectrum of control peptides, (4) further optimized the sample cleanup using a method with magnetic beats allowing for high throughput measurements, (5) performed extensive stability tests (6) increased the sensitivity of the method, (7) studied a large number of human subjects, including a distinct CKD population (8) measured samples from apelin transfected human embryonic cells, a “positive control” sample that has not been studied before.

Despite all these improvements, we could not detect any of a wide range of apelin isoforms in the plasma of healthy volunteers (n = 16) and CKD patients (n = 4) by HPLC–MS/MS.

The various molecular forms of apelin have a very short half-life. In general, protein degradation can be slowed by cooling, the use of protease inhibitors and acidification. While individual components of this strategy have been applied, the effect on apelin degradation remains largely unknown and no uniformly accepted protocol exist. The type of tube and the acidification of plasma could have an important effect on the proteins in blood samples^22^. The effect of the P100 protease inhibitor cocktail on apelin remained unstudied. Our general conclusion is that, for the determination of synthetic apelin peptides, it is best to process the samples at 4 °C and acidify plasma as quickly as possible to pH 2.5. Because the P100 tube does not significantly increase stability, while it potentially exerts a negative influence on the HPLC–MS/MS method by ion suppression, the K_2_EDTA blood tube is best suited for HPLC–MS/MS measurements.

Earlier experiences with MS based methods for detection and quantification of apelin in human samples had been disappointing^8,12^. While we optimized the methodology, our HPLC–MS/MS method was also not able to detect apelin isoforms in human plasma of 16 healthy volunteers and 4 CKD patients. However, a previously validated RIA method^9,14,15^, was able to detect apelin immunoreactivity corresponding in majority to apelin-17 and pyroglutamyl apelin-13 and to a much lesser extent to apelin-36 (Table 7). Several hypotheses can be raised to explain the inability of detecting measurable quantities of the apelin isoforms by our HPLC–MS/MS method.

First, it could be that the endogenous peptides were lost during sample preparation because of adherence to the plastic tubing despite using low protein binding material and QuanRecovery plates. To rule this out, we remeasured K_2_EDTA pH2.5 plasma samples of two healthy volunteers and two CKD patients with and without the addition of a final concentration of 1% bovine serum albumin (BSA), as the latter should prevent adherence of the culprit analytes to the plastics. Still, no apelin could be detected.

A second explanation could be that the current MS method is not sensitive enough. However, in comparison with Mesmin et al.^11^ (who were also unable to detect endogenous peptides) and despite the fact that only half of the sample volume was needed, we reached a better sensitivity for the small peptides apelin-17, 13, pyr-13 and 12 and approximately the same sensitivity for apelin-36. This increase in sensitivity for the smaller isoforms is probably due to better recoveries obtained after sample preparation. Compared to Zhen et al.^8^, we obtain about the same sensitivities (Table 8), while only half the sample volume is required. They were able to detect apelin apelin-pyr-13 in 5 of 6 human plasma samples (range 7.7–23.3 pg/ml), while they were unable to detect apelin-36, apelin-17, and apelin-13. While the sensitivity of the current method together with an improved sample stability at pH 2.5 and the use of low protein bound storage tubes should allow for the detection of such low quantities, we were unable to do so. Our findings are in line with a recent paper^12^ that measured apelin-pyr-13 in vivo after infusing intact apelin-pyr-13 in humans using LC-MSMS, but was also unable to detect apelin-pyr-13 in the plasma of 6 healthy volunteers before infusion. However, we should note the relatively high LOQ of 1 ng/ml in this paper.

**Table 8:** Comparison of MS method sensitivities.

Peptide	Current LLOQ (pg/ml)	Zhen et al.^8^ LLOQ (pg/ml)	Nyimanu et al. LLOQ^12^ (pg/ml)
Apelin-36	80.0	100.0	NA
Apelin-17	2.0	25	NA
Apelin-13	2.0	1.59	NA
Apelin-pyr-13	8.0	6.25	1000
Apelin-12	2.0	3.18	NA

While we were unable with the current MSMS method to detect apelin in biological samples, we did detect apelin with a commercial immunoassay (EKE-057-15, Phoenix Pharmaceuticals) in the plasma of 4 healthy volunteers at concentrations reaching a few 100 pg/ml (data not shown). By far the most widely published commercial immunoassays for the measurement of apelin are from Phoenix Pharmaceuticals. All these kits are based on the principle of competitive enzyme immunoassay (EIA). The company offers kits for the determination of apelin-36 or -12. Apelin-36 kits (EK or EKE-057-15) are said to have a 100% cross reactivity with human apelin-12 and -13. The primary antibody is a rabbit polyclonal that had been raised against a fully synthetically peptide corresponding to human apelin-36. In contrast, the apelin-12 kits (EK or EKE-057-23) contain a primary antibody raised against a synthetically peptide corresponding to human apelin-12. Again, cross reactivity with apelin-36 and -13 is indicated.

Zhen et al. found apelin-pyr-13 in concentrations ranging from 49.3 to 273 pg/ml with a Phoenix EIA kit while the MS-measured apelin was 4- to 20-fold lower (range 7.7–23.3 pg/ml) without any clear correlation. It could be that these immunoassay methods are more sensitive. Indeed, the cross reactivity against apelin fragments is not trivial and it has to be concluded that it is unknown what fraction of apelin fragments are measured by individual immunoassays, making it ad minimum (assuming that only apelin fragments are recognized) impossible to compare results of different kits. However, the fact that potentially many isoforms and fragments are recognized could increase the sensitivity compared to MS-based methods by measuring effectively a ‘total apelin pool’. Of note, the primary antibodies of these kits are rabbit polyclonal. This is important, because only 0.5–5% of the antibodies in a polyclonal reagent bind to the putative target and functionality varies from batch to batch^23^. Until recombinant antibodies defined by their sequences are validated, great caution should be taken^23,24^.

MS methods are very specific. Due to specificity for individual isoforms, lower concentrations than a ‘total apelin pool’ are expected. In addition, the peptides have multiple charges, and because we choose to detect only 1 charge with 2 m/z transitions, an inherent loss of sensitivity occurs. More charges occur in larger peptides, visible in our calibration curves information (Table 8), where the larger peptides have higher LLOQ and LOD. Switching to a nano or microscale based liquid chromatography technique with MSMS could increase the sensitivity to selectively detect the individual isoforms separately.

Rapid peptide degradation or modification, despite optimal sample preparation and storage, could be another reason for the detection problem. While we obtained satisfactory stability of synthetic apelin isoforms, we cannot rule out higher lability of biological isoforms. A recent mass spectrometer paper confirmed the lability of synthetic peptides in the humans in vivo^12^. Using full scan LC–MS/MS with the orbitrap mass spectrometer and metabolite identification with the Qualbrowser software package (https://www.thermofisher.com/order/catalog/product/OPTON-30965), they were able to detect several C- and N-terminally cleaved fragments of apelin-pyr-13. Since we did not perform such an analysis, we cannot rule out similar degradation patterns for the synthetic or endogenous peptides. When spiking with individual synthetic isoforms during stability tests, we had expected that degradation of the long peptides would result in an increase in the shorter isoforms, but this was not the case. However, after spiking synthetic apelin-13, we clearly saw apelin-pyr-13 appear after 60 min. This was minimal with the K_2_EDTA non-acidified condition, more at pH 4.5 (± 3× more than non-acidified), and most at pH 2.5 (± 25% more than at pH4.5). Similar observations were made with the P100 tube. This indicates that at least synthetic apelin-13 not only decreased in the concentration due to degradation but also due to post translational modifications (PTM) in the plasma. This raises the interesting hypothesis that in vivo apelin isoforms undergo post translational modifications. For example, Mesmin et al.^11^ and Zhen^8^ et al. had already suspected a process of oxidation, but could not demonstrate this using both targeted and untargeted approaches. As the detection with mass spectrometry is very specific and based on synthetic peptide standards without potential posttranslational modifications, it could be that PTM into other peptides that are not detected explains our inability to measure endogenous apelin fragments. With this in mind, our finding in HEK cells is intriguing. As these are human cells, we could expect that intracellular posttranslational modifications are reproduced*.* However, this model does not take into account any potential extracellular processes. When we studied apelin transfected HEK cells, we could find a 2.5× increase in apelin-36 but not in other isoforms. At the same time, no apelin isoforms were detected in the cell culture medium. One explanation could be that apelin undergoes further modifications before secretion. It would be extremely interesting to use human transfected cell models to identify potential metabolites using full scan LC–MS and software analysis in order to detect and identify potential metabolites that could then be detected in plasma samples.

Apelin could be detected in 10 plasma samples of healthy volunteers using a validated RIA-detection method^9,13–15^. While the addition of a chromatographic separation step increases the specificity for isoforms, and the relative specificity of the antibodies has been confirmed, there is also important cross reactivity between isoforms and a certain affinity for smaller degradation products with this method. Nevertheless, based on literature data and our own findings, we must conclude that RIA detection, is currently the best available technique to quantify total apelin with relative specificity.

In summary, from the current work we conclude that a state of the art HPLC–MS/MS method was unable to detect any individual apelin isoform in human plasma. Hence, while the comprehension of isoforms-specific biological effects increases, our current knowledge on specific circulating isoforms remains inadequate. Further research is needed to develop sensitive and specific methods for the measurement of the apelinergic ligands apelin. Currently, the combination of HPLC separation and detection with relatively selective antibodies is the best alternative for the measurement of apelin isoforms.

## Supplementary Information

Supplementary Information.

## Data Availability

The datasets generated during and/or analyzed during the current study are available from the corresponding author on reasonable request.

## References

[1] Janssens P (2021). The emerging role of the apelinergic system in kidney physiology and disease. Nephrol. Dial. Transplant..

[2] Pauli A (2014). Toddler: An embryonic signal that promotes cell movement via Apelin receptors. Science.

[3] Chng SC, Ho L, Tian J, Reversade B (2013). ELABELA: A hormone essential for heart development signals via the apelin receptor. Dev. Cell.

[4] Zeng XX, Wilm TP, Sepich DS, Solnica-Krezel L (2007). Apelin and its receptor control heart field formation during zebrafish gastrulation. Dev. Cell.

[5] Ho L (2015). ELABELA is an endogenous growth factor that sustains hESC self-renewal via the PI3K/AKT pathway. Cell Stem Cell.

[6] O'Carroll AM, Lolait SJ, Harris LE, Pope GR (2013). The apelin receptor APJ: Journey from an orphan to a multifaceted regulator of homeostasis. J. Endocrinol..

[7] Wang Z (2015). Elabela-apelin receptor signaling pathway is functional in mammalian systems. Sci. Rep..

[8] Zhen EY, Higgs RE, Gutierrez JA (2013). Pyroglutamyl apelin-13 identified as the major apelin isoform in human plasma. Anal. Biochem..

[9] Azizi M (2008). Reciprocal regulation of plasma apelin and vasopressin by osmotic stimuli. J. Am. Soc. Nephrol..

[10] Shin K, Kenward C, Rainey JK (2017). Apelinergic system structure and function. Compr. Physiol..

[11] Mesmin C, Dubois M, Becher F, Fenaille F, Ezan E (2010). Liquid chromatography/tandem mass spectrometry assay for the absolute quantification of the expected circulating apelin peptides in human plasma. Rapid Commun. Mass Spectrom..

[12] Nyimanu D (2019). Development and validation of an LC-MS/MS method for detection and quantification of in vivo derived metabolites of [Pyr(1)]apelin-13 in humans. Sci. Rep..

[13] Maguire JJ, Kleinz MJ, Pitkin SL, Davenport AP (2009). [Pyr1]apelin-13 identified as the predominant apelin isoform in the human heart: Vasoactive mechanisms and inotropic action in disease. Hypertension.

[14] De Mota N (2004). Apelin, a potent diuretic neuropeptide counteracting vasopressin actions through inhibition of vasopressin neuron activity and vasopressin release. Proc. Natl. Acad. Sci. U.S.A..

[15] Reaux A (2001). Physiological role of a novel neuropeptide, apelin, and its receptor in the rat brain. J. Neurochem..

[16] Blanchard A (2013). An abnormal apelin/vasopressin balance may contribute to water retention in patients with the syndrome of inappropriate antidiuretic hormone (SIADH) and heart failure. J. Clin. Endocrinol. Metab..

[17] Brash L (2018). Short-term hemodynamic effects of apelin in patients with pulmonary arterial hypertension. JACC Basic Transl. Sci..

[18] Japp AG (2008). Vascular effects of apelin in vivo in man. J. Am. Coll. Cardiol..

[19] Yi J, Kim C, Gelfand CA (2007). Inhibition of intrinsic proteolytic activities moderates preanalytical variability and instability of human plasma. J. Proteome Res..

[20] Goebel-Stengel M, Stengel A, Tache Y, Reeve JR (2011). The importance of using the optimal plasticware and glassware in studies involving peptides. Anal. Biochem..

[21] Armbruster DA, Tillman MD, Hubbs LM (1994). Limit of detection (LQD)/limit of quantitation (LOQ): Comparison of the empirical and the statistical methods exemplified with GC-MS assays of abused drugs. Clin. Chem..

[22] Cruickshank-Quinn C (2018). Impact of blood collection tubes and sample handling time on serum and plasma metabolome and lipidome. Metabolites.

[23] Bradbury A, Pluckthun A (2015). Reproducibility: Standardize antibodies used in research. Nature.

[24] Baker M (2015). Reproducibility crisis: Blame it on the antibodies. Nature.

[25] Adam F (2016). Apelin: An antithrombotic factor that inhibits platelet function. Blood.

[26] Soulet F (2020). ELA/APELA precursor cleaved by furin displays tumor suppressor function in renal cell carcinoma through mTORC1 activation. JCI Insight..

[27] Wang W (2016). Angiotensin-Converting Enzyme 2 Metabolizes and Partially Inactivates Pyr-Apelin-13 and Apelin-17: Physiological effects in the cardiovascular system. Hypertension.

[28] McKinnie SMK (2017). Synthetic modification within the "RPRL" region of apelin peptides: Impact on cardiovascular activity and stability to neprilysin and plasma degradation. J. Med. Chem..

[29] Fischer C (2019). Plasma kallikrein cleaves and inactivates apelin-17: Palmitoyl- and PEG-extended apelin-17 analogs as metabolically stable blood pressure-lowering agents. Eur. J. Med. Chem..

[30] Kehoe K (2016). Prolyl carboxypeptidase purified from human placenta: Its characterization and identification as an apelin-cleaving enzyme. Biochim. Biophys. Acta.

