# Supplementary material for: On Methods for the Measurement of the Apelin Receptor Ligand Apelin
Source: Sci Rep. 2022 May 11;12:7763. doi: 10.1038/s41598-022-11835-z (PMC9095593; doi:10.1038/s41598-022-11835-z)
Supplement: Supplementary file 1 — Supplementary Information. [file 41598_2022_11835_MOESM1_ESM.pdf]

SUPPLEMENTARY INFORMATION

On Methods for the Measurement of the Apelin Receptor Ligand Apelin

Authors:

Peter Janssens, Henriette de Loor, Jean-Paul Decuypere, Rudi Vennekens, Catherine Llorens-Cortes, Djalila Mekahli, Bert Bammens

Contents :

Supplementary Method

Supplementary Figure 1, 2 & 3

Supplementary Method

The final chosen solving solutions of MilliQ water/acetonitrile (90/10) + 0.1% formic acid was concluded after the following test: A fixed concentration was prepared in the following conditions, pure MilliQ water, MilliQ water + 1% formic acid, MilliQ water + 0.1% formic acid, MilliQ water/acetonitrile (95/5) + 0,1% formic acid, MilliQ water/acetonitrile (90/10) + 0,1% formic acid and MilliQ water/acetonitrile (90/10) + 1% formic acid. These solutions were injected 3 times directly without sample preparation on the HPLC-MS/MS, mean areas were compared, on day 0 and 1, 2 and 6 days after storage in the refrigerator at 4°C (supplementary figure 1). In addition, the area was compared after none, 1x, 2x and 3x freeze/thaw cycling (FTC) at -80°C of the solutions (supplementary figure 2).

Note that for apelin-36, acidification without the presence of acetonitrile is not stable at 4° nor for the FTC. For apelin-17, -13 and -12, all the tested conditions were satisfactory. For apelin-pyr-13, acidification makes a clear difference. For apela-32 and -21 there is a clear benefit of 10% acetonitrile after FTC. Both conditions MilliQ water/acetonitrile (90/10) + 0,1% formic acid and MilliQ water/acetonitrile (90/10) + 1% formic acid were good for stability at 4° and FTC. We ultimately opted for the 0.1% formic acid with MilliQ water/acetonitrile (90/10) to counteract any chance of artificial formylation.

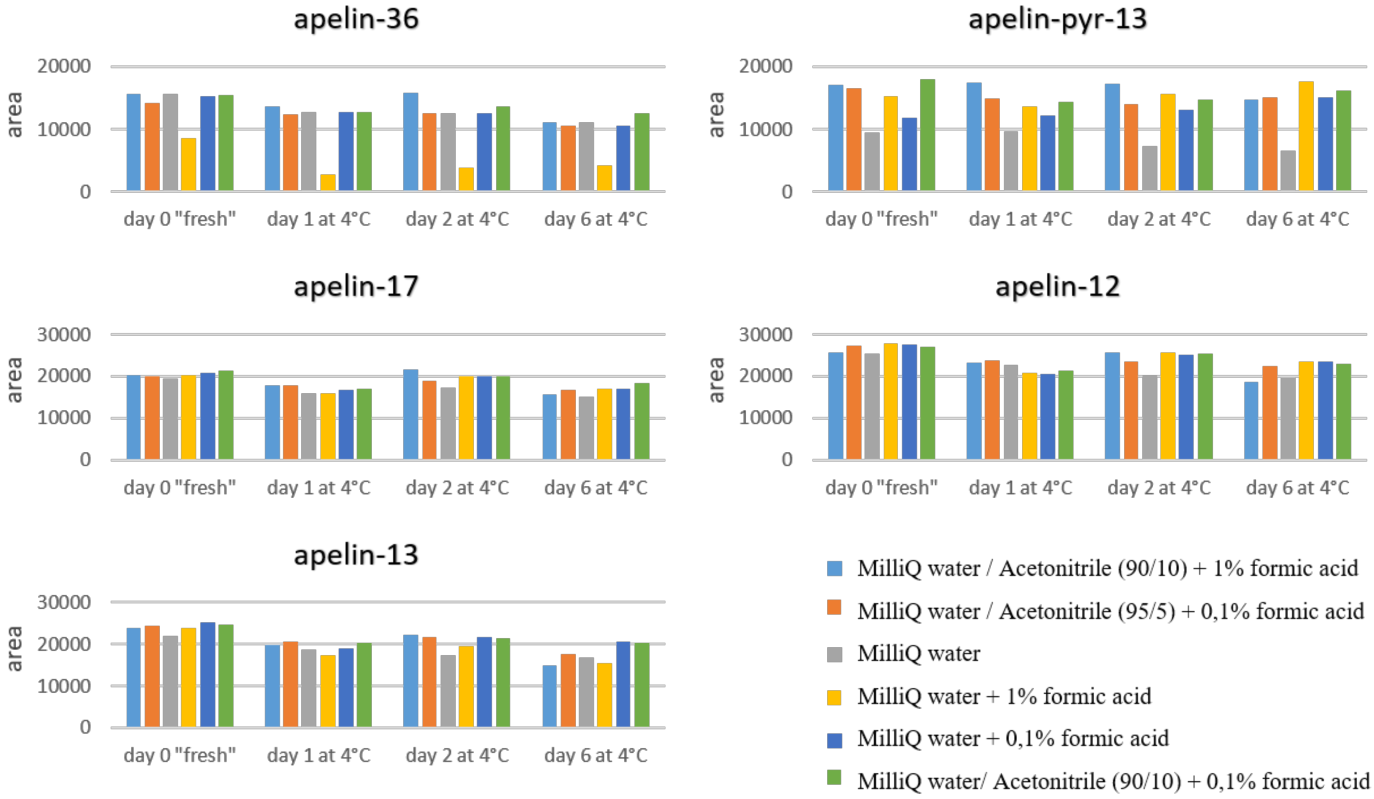

Supplementary figure 1: stability apelin/apela solutions at 4°C

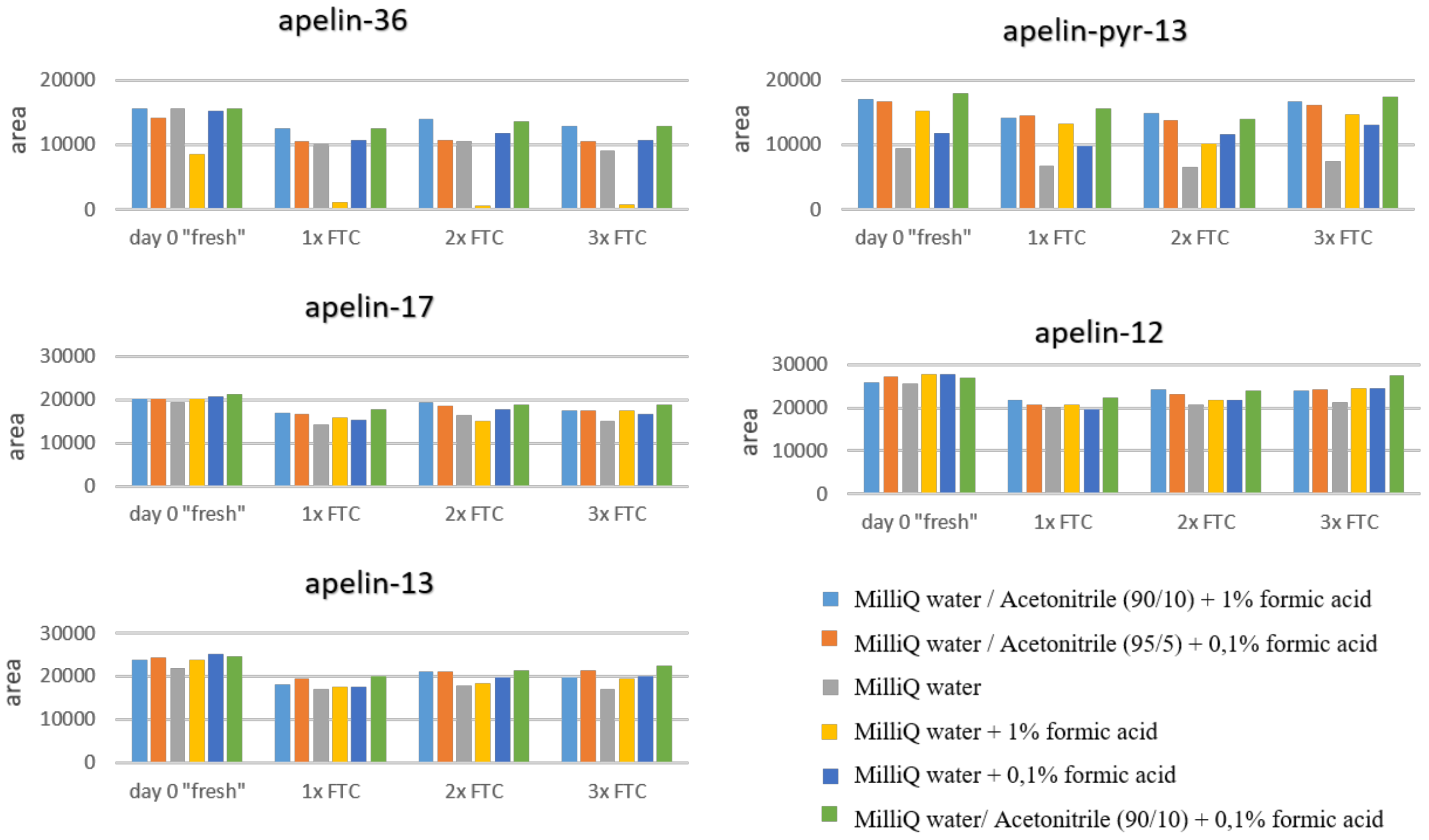

Supplementary figure 2: Freeze/thaw cycle (FTC) stability apelin/apela solutions

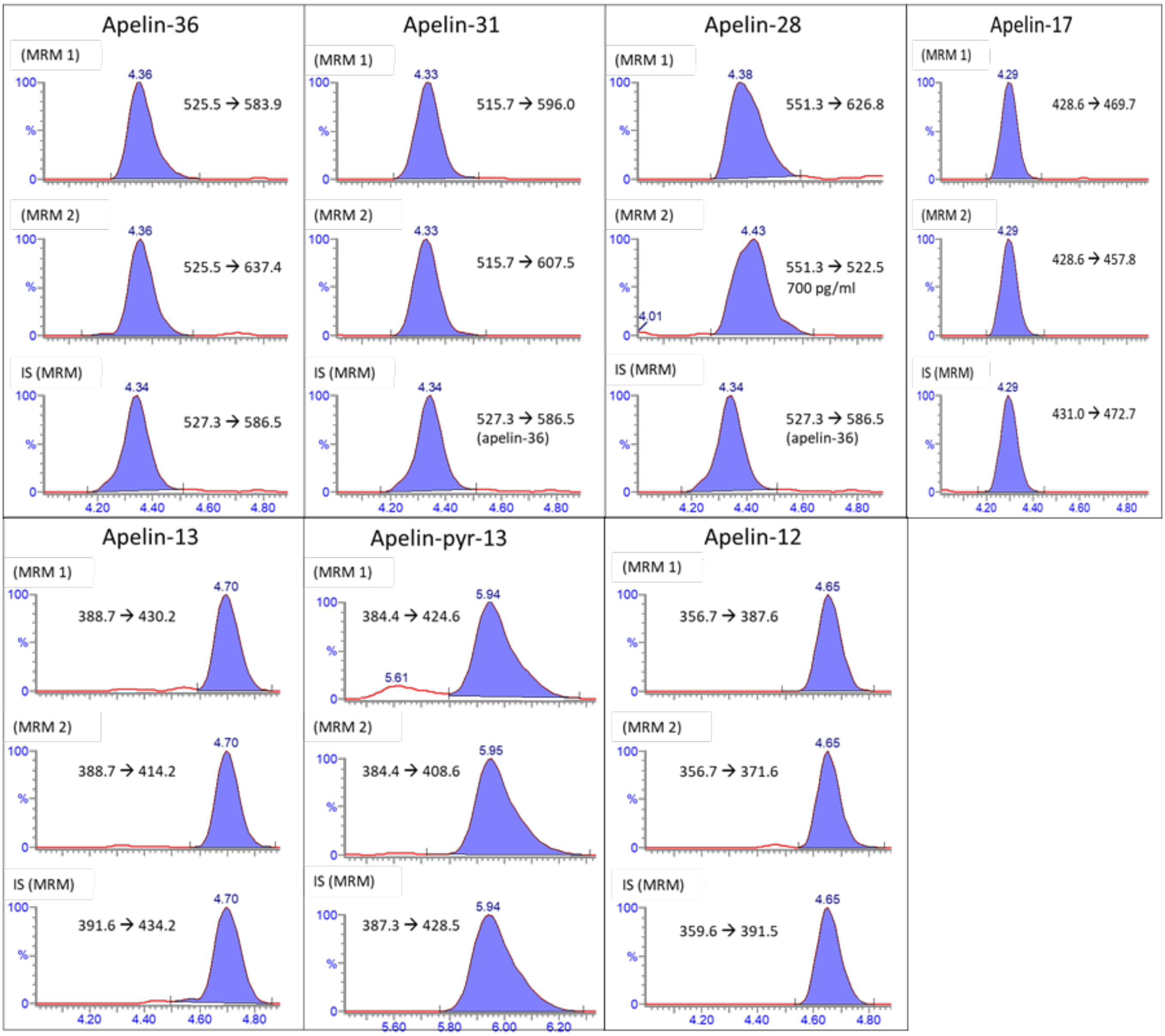

Supplementary figure 3: Typical chromatograms of synthetic spiked isoform of apelin on healthy K2EDTA plasma pool at pH 4.5 (free of endogenous apelin). The MRM transition and retention time are mentioned for each. The spiked concentration is 700 pg/ml for apelin-36, -31 and -28, and 250 pg/ml for apelin-17, 13, -pyr-13 and -12.
